# Serum Cytokine Profile in a Patient Diagnosed with Dysferlinopathy

**DOI:** 10.1155/2017/3615354

**Published:** 2017-04-13

**Authors:** Svetlana F. Khaiboullina, Ekaterina V. Martynova, Sergey N. Bardakov, Mikhail O. Mavlikeev, Ivan A. Yakovlev, Arthur A. Isaev, Roman V. Deev, Albert A. Rizvanov

**Affiliations:** ^1^Institute of Fundamental Medicine and Biology, Kazan Federal University, Kazan, Russia; ^2^University of Nevada, Reno, Reno, NV, USA; ^3^Kirov Military Medical Academy, Saint Petersburg, Russia; ^4^Human Stem Cell Institute, Moscow, Russia; ^5^Ryazan State Medical University Named after Academician I. P. Pavlov, Ryazan, Russia

## Abstract

Limb-girdle muscular dystrophy type 2 (LGMD2B) is a mild form of dysferlinopathy, characterized by limb weakness and wasting. It is an autosomal recessive disease, with currently 140 mutations in the LGMD2B gene identified. Lack of functional dysferlin inhibits muscle fiber regeneration in voluntary muscles, the main pathological finding in LGMD2B patients. However, the immune system has been suggested to contribute to muscle cell death and tissue regeneration. Serum levels of 27 cytokines were evaluated in a dysferlinopathy patient. Levels of 8 cytokines differed in patient serum compared to controls. Five cytokines (IL-10, IL-17, CCL2, CXCL10, and G-CSF) were higher while 3 were lower in the patient than in controls (IL-2, IL-8, and CCL11). Together, these data on serum cytokine profile of this dysferlinopathy patient suggest immune response activation, which could explain leukocyte infiltration in the muscle tissue.

## 1. Introduction

Dysferlin, also known as dystrophy-associated fer-1-like protein, is involved in skeletal muscle repair. Dysferlinopathy is a rare genetic disease, with an incidence rate estimated to be between 1 in 100,000 and 1 in 200,000 [1]. Limb-girdle muscular dystrophy type 2 (LGMD2B) is a mild form of dysferlinopathy characterized by weakness and wasting in the skeletal muscles of the limbs and limb girdles, while cardiac and respiratory muscles are usually not involved. Patients become confined to a wheelchair on average 10–20 years after onset.

Dysferlin plays a role in cell membrane repair. Muscle cell membrane is frequently damaged, thus requiring high levels of dysferlin expression to restore its integrity. In LGMD2B, a mutation within the DYSF gene abrogates protein expression and is believed to cause the muscle dystrophy [2]. Additionally, damage to muscle causes local inflammation, evidence of which is often found in LGMD2B histology. Therefore, inflammation may play a role in the pathogenesis of LGMD2B. In this case report, we present the serum cytokine profile of a patient diagnosed with LGMD2B.

Inflammation plays a role in the pathogenesis of skeletal muscle dysfunction. For example, the pathogenesis of muscle necrosis in dermatomyositis was associated with activation of proinflammatory cytokines [3, 4]. Also, inflammasome upregulation was demonstrated in dysferlin-deficient muscles, suggesting activation of proinflammatory cytokines including IL-1*β* and IL-18 [5]. Additionally, cells expressing proinflammatory cytokines were detected in muscle biopsies of patients with idiopathies [6, 7]. Proinflammatory cytokines can trigger leukocyte migration including CD8^+^ T-lymphocytes [8]. Activated CD8+ T-lymphocytes infiltrating muscle tissue can release perforin and granzyme, causing muscle degeneration and necrosis. However, little is known about cytokine activation in dysferlinopathy.

## 2. Materials and Methods

### 2.1. Patient

The patient, a 45-year-old male, was diagnosed with dysferlinopathy in 2002. The Institutional Review Board of the Kazan Federal University approved this study and informed consent was obtained from the patient according to the guidelines approved under this protocol (article 20, Federal Law “Protection of Health Rights of Citizens of Russian Federation” N323- FZ, 11.21.2011). Clinical, genealogical, and neurological data were collected. A tissue biopsy obtained from the right m. tibialis anterior was cryopreserved. Cryosections (5 *μ*m) were stained with hematoxylin to identify fiber types. The expression of dysferlin was assessed using immunoblotting and immunohistochemical staining with anti-dysferlin antibodies (NCL-Hamlet, Novocastra) (performed by Dr. S. Thiele, Ludwig Maximilian University of Munich, Germany).

### 2.2. Cytokine Analysis

Serum cytokines were analyzed using Bio-Plex Pro Human 27-Plex Panel (Bio-Rad, Hercules, CA, USA) multiplex magnetic bead-based antibody detection kits following the instructions. Serum aliquots (50 *μ*L) were collected from the dysferlinopathy patient and six controls. A minimum of 50 beads per analyte were acquired. Median fluorescence intensities were measured using a Luminex 200 analyzer. Data collected were analyzed with MasterPlex CT control software and MasterPlex QT analysis software (Hitachi Software, San Bruno, CA, USA). Standard curves for each analyte were generated using standards provided by the manufacturer.

## 3. Results and Discussion

### 3.1. Clinical and Genealogical Analysis

Proband U (III:4) (male, 45 years old) complained of weakness in the legs and distal parts of the arms, impaired gait, and a decreased muscle volume in the lower parts of the legs, thighs, and forearms. Distal Miyoshi myopathy (MM, OMIM # 254130) was diagnosed.

**Figure 1:**
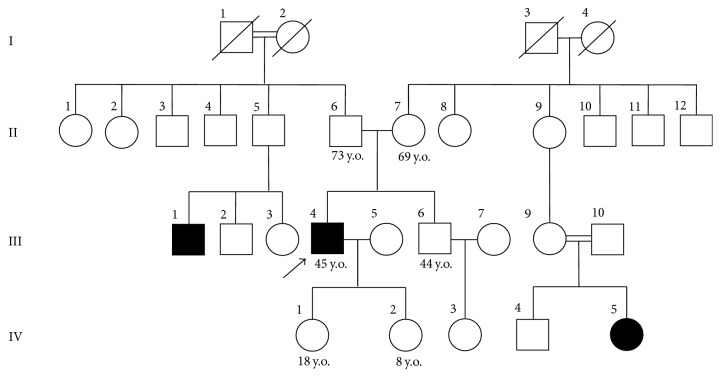
Genealogy of Proband U (III:4). An arrow indicates the Proband: black, affected family member; white, healthy family member. I, II, III, and IV are generations within the Proband's family. III:1, manifestation at the age of 21 years (muscle weakness in the distal regions of the lower extremities). III:4, manifestation at the age of 26 years (muscle weakness in the posterior group of the lower leg muscles). IV:5, manifestation at the age of 21 years (muscle weakness in the distal regions of the lower extremities).

The Proband's parents are ethnic Mountain Jews (Tat people), descendants of an isolated settlement in the Republic of Dagestan, Russia. Both parents are healthy and have never complained from muscle weakness. Two intermarriages and three cases of progressive muscle dystrophies with similar signs (III:4-Proband; III:1; IV:5) were identified in the family pedigree. The Proband has two daughters, aged 18 (IV:1) and 8 (IV:2), who currently have no clinical signs of the disease. Autosomal recessive inheritance of progressive muscle dystrophy was suggested (Figure 1).

### 3.2. Medical History

The Proband was born in term to clinically healthy parents without prenatal pathology. Characteristic gait including high foot rising was noted for the first time at the age of 23. Three years later, a significant weakness in m. gastrocnemius was noticed, preventing him from standing up on his toes and jumping. During the next 7 years (to the age of 30), a slow progressive weakness in the distal region of the lower extremities was observed. Since the age of 32, he has experienced weakness in the proximal region of the lower extremities and used a walking stick. From the age of 36, due to occasional falls, he has constantly used a walking stick. At 39, the patient began experiencing significant difficulties in getting up from a seated position and walking on an inclined surface and fatigue in his arm muscles. From the age of 44, the patient started experiencing rapid fatiguing in the lumbar extensor muscles while sitting.

### 3.3. Histopathological Analysis

There were moderate diffuse interstitial changes in the form of peri- and endomysial fibrosis in muscle tissue. Additionally, a small number of interfascicular aggregates of adipose tissue were detected. In some fields, prominent connective tissue septa were found between muscle fibers, presenting a pseudolobular appearance. In cross sections, all muscle fibers were round in shape and varied in size, with some fibers appearing hypotrophic and rarely others appearing hypertrophic. Dystrophic changes in the muscle fibers were categorized as hyaline degeneration. Additionally, in some fields, early muscle fiber necrosis was detected with distinct macrophage infiltration. Blood vessels remained unaffected. Analysis of ATPase activity demonstrated muscle fiber mosaicism, with type II fibers more variable in size than type I. The number of type I fibers was higher than type II, indicating a moderate degenerative myopathy. Immunoblotting and immunohistochemical staining confirmed that there was lack of dysferlin expression in skeletal muscles of the patient.

### 3.4. Neurological Examination

The patient was fully conscious and conversant. Active movements that were limited in the distal regions of the lower extremities included flexion, abduction, and adduction in the feet at 0 degrees. The patient had Trendelenburg gait with elements of circumduction to compensate for a limited ability to raise the foot. Muscle strength was characterized as distal-proximal myogenic tetraparesis predominantly in the lower extremities (Table 1). The results of hand dynamometry were 36/36 kg. Muscles of the shoulder girdle exhibited moderate amyotrophia, which was more severe in the lower third of the medial portion of the forearm. Moderate atrophy was revealed in muscles of the pelvic girdle. Severe amyotrophic changes were found in the anterior and medial groups of the thigh muscles, mostly in the lower third. The lower leg muscles, predominantly the posterior group, exhibited the most severe atrophies. Compensatory hypertrophy was detected in the short toe flexor muscle.

### 3.5. Genetic Testing

A genetic analysis performed using PCR revealed a frameshift mutation c.2779delG (Ala927LeufsX21) in exon 26 of the gene* dysf*, which has been previously associated with the disease.

### 3.6. Cytokine Analysis

Out of 27 cytokines studied, 8 cytokines differed in this dysferlinopathy patient compared to healthy controls. Serum levels of seven cytokines, IL-10, IL-17, CCL2, CCL5, CXCL10, G-CSF, and GM-CSF, were higher in the patient than in controls, while levels of IL-2, IL-8, CCL11, and VEGF were lower (Table 2).

Based on the function, these cytokines could be divided into three categories: (1) leukocyte chemoattractants and proliferation signals, (2) regulators of angiogenesis, and (3) immunoregulatory cytokines.

CXCL10, CCL2, CCL5 G-CSF, and GM-CSF are in the chemoattractant and proliferation signal group. The serum cytokine profile suggests activation and tissue migration of mononuclear leukocytes (CXCL10, CCL2, and CCL5) as well as granulocytes (CCL5 G-CSF, and GM-CSF). Additionally, increased IL-17 levels in the patient serum indicate upregulation of the Th17 type immune response, which is known to be pathogenic and is often associated with tissue damage [9].

Serum G-CSF and CM-CSF were upregulated in the patient serum relative to controls. G-CSF activates neutrophils and initiates proliferation and differentiation into mature granulocytes. Similarly, GM-CSF is a potent growth factor stimulating stem cells to differentiate into neutrophils and monocytes [10]. Neutrophil infiltration can cause inflammation and tissue damage suggesting that tissue damage found in muscle tissue is, in part, caused by local inflammation and neutrophil activation. Additionally, chemokines that promote migration of monocytes and neutrophils are upregulated in the serum of this patient. Therefore, we proposed that the pathogenesis of dysferlinopathy is due to neutrophil and monocytes proliferation and tissue migration. It should be noted that the serum level of IL-8, the prototype neutrophil chemoattractant, was downregulated in the patient's serum. This was rather unexpected; however, IL-8 may play a role locally in muscle tissue that is not reflected in the serum level.

Eosinophil infiltration has been reported in muscle tissue of patients with a range of myopathies [11], although there was a lack of correlation between the number of eosinophils and the extent of myopathy. Several cytokines regulate eosinophil chemotaxis, including CCL11 [12, 13]. We found a lower serum level of CCL11 in the patient compared to controls. This suggests that eosinophil migration into muscle tissue is regulated by a different set of cytokines, produced locally within the tissue. For example, GM-CSF and CCL5 are potent chemoattractants and stimulators of eosinophil proliferation [14–16].

The second group of cytokines includes VEGF, a potent angiogenic factor. Serum VEGF was lower in the dysferlinopathy patient relative to controls. In addition to angiogenesis, VEGF is known to be a neurotrophic factor, protecting neurons from hypoxia. For example, VEGF upregulates myoglobin levels in ischemic skeletal muscle, resulting in improved muscle oxygenation and survival [17]. Also, VEGF has been shown to regulate neurogenesis, by promoting neuronal migration and survival [18]. Low serum level of VEGF may explain muscle tissue repair defects in dysferlinopathy patients. We suggest that low VEGF levels affect vascularization of skeletal muscle tissue, leading to hypoxia.

Finally, the third group included cytokines (IL-2 and IL-10) with immunoregulatory function. Low levels of IL-2 were found in the serum of the dysferlinopathy patient. IL-2 is a pleiotropic regulatory cytokine involved in the regulation of T-cell differentiation [19]. It has been suggested that IL-2 modulates the signaling pathway essential for differentiation into different types of T-helper cells [20]. Therefore, it was proposed that, depending on the cytokine milieu, IL-2 may act as a master regulator influencing the selection of the T-lymphocyte differentiation pathway. Increased IL-10 and IL-17 levels in dysferlinopathy serum suggest activation of Th17 and Treg lymphocytes. However, low serum IL-2 may indicate that this cytokine is not contributing to lymphocyte differentiation.

## 4. Conclusions

Increased serum chemokines, CCL5, CXCL10, G-CSF, and GM-CSF, may explain leukocyte infiltration found in the patient's muscle tissue. Decreased serum level of VEGF could reflect the tissue hypoxia and inhibition of angiogenesis in muscle biopsies. The cytokine profile suggests the role of Th17 in the pathogenesis of dysferlinopathy as increased IL-17 and decreased IL-10 levels were found in the patient's serum. Therefore, data suggest that the immune mechanism contributes to the muscle tissue damage found in the dysferlinopathy patient.

## Figures and Tables

**Table 1:** Clinical characteristics of Proband (III:4) skeletal muscles.

Muscle name	Muscle strength (MRC, score)	Intensity of atrophy
Deltoid	4	No atrophy observed
Biceps brachii	4	No atrophy observed
Triceps brachii	5	No atrophy observed
Pectoralis major	4	Severe atrophy of the middle portion of the right pectoralis major
Extensor digitorum	5	Normal volume
Flexor digitorum superficialis	5	Atrophy more pronounced in medial bundles
Flexor digitorum profundus	4	Atrophy of the ulnar portion in the lower third of the forearm (flexors of fingers V and IV) is more pronounced
Thenar group	5	No atrophy observed
Hypothenar group	5	No atrophy observed
Trapezius	4	No atrophy observed
Latissimus dorsi	4	No atrophy observed
Erector spinae	4	No atrophy observed
Glutei	4	Moderate atrophy
Quadriceps femoris	3.5	Severe atrophy in the medial and lateral vastus muscles
Biceps femoris	3.5	Severe atrophy
Adductor major	2.5	Severe atrophy
Tractus iliotibialis (tensor fascie latae)	3.5	Moderate atrophy
Gastrocnemius medial/lateral	1	Severe atrophy
Soleus	1	Severe atrophy
Tibialis anterior	2	Moderate atrophy
Extensor digitorum longus	3	Moderate atrophy
Extensor digitorum brevis	4	Hypertrophy

**Table 2:** Analysis of patient and controls cytokine profile.

Analyte	Control (*n* = 6)	Patient
Immunoregulatory		
IL-1b	19.6 ± 4.9	18.1
IL-1RA	376.2 ± 162.7	291.7
IL-2	129 ± 17.9	78^*∗*^
IL-4	78.8 ± 5.0	87
IL-5	1.5 ± 0.3	0.7 ± 0.5
IL-6	67.3 ± 21.0	46.9
IL-7	2.5 ± 1.3	3.5 ± 1.5
IL-9	80.4 ± 9.9	89
IL-12 (p70)	31.1 ± 1.0	23
IL-10	31.7 ± 3.7	95^*∗*^
IL-13	59.3 ± 5.4	35
IL-15	131.3 ± 34.3	160.1
IL-17	3826.8 ± 367.7	5483^*∗*^
CCL11	4314.4 ± 1320.2	1984^*∗*^
IFN-*γ*	2960.3 ± 468.7	2391

Leukocyte chemoattractants		
CCL2	444.9 ± 132.9	926.2^*∗*^
CCL3	23.3 ± 2.4	18
CCL4	615.2 ± 147.8	453.4
CCL5	40267.4 ± 1767.8	60422.1
CXCL10	7446.1 ± 1865.6	9446.9^*∗*^
G-CSF	111.9 ± 20.1	202.9^*∗*^
GM-CSF	80.7 ± 22.8	56
IL-8	370.6 ± 170.7	37.3^*∗*^
TNF-*α*	229.2 ± 22.6	210

Angiogenesis		
VEGF	2367.1 ± 705.7	856
PDGF-BB	34535.4 ± 2952.9	39325.6
bFGF	2.4 ± 1.4	1.8 ± 0.9

^*∗*^Values differ considerably from that in the control.
